# Autologous and Heterologous Minor and Major Bone Regeneration with Platelet-Derived Growth Factors

**DOI:** 10.3390/jfb16010016

**Published:** 2025-01-09

**Authors:** Gianna Dipalma, Angelo Michele Inchingolo, Valeria Colonna, Pierluigi Marotti, Claudio Carone, Laura Ferrante, Francesco Inchingolo, Andrea Palermo, Alessio Danilo Inchingolo

**Affiliations:** 1 Department of Interdisciplinary Medicine, University of Bari “Aldo Moro”, 70121 Bari, Italy; giannadipalma@tiscali.it (G.D.); angeloinchingolo@gmail.com (A.M.I.); or valeria.colonna@uniba.it (V.C.); or pierluigi.marotti@uniba.it (P.M.); or claudio.carone@uniba.it (C.C.); lauraferrante79@virgilio.it (L.F.); ad.inchingolo@libero.it (A.D.I.); 2 Department of Experimental Medicine, University of Salento, 73100 Lecce, Italy; andrea.palermo@unisalento.it

**Keywords:** minor bone regeneration, major bone regeneration, autologous, heterologous, growth factor

## Abstract

Aim: This review aims to explore the clinical applications, biological mechanisms, and potential benefits of concentrated growth factors (CGFs), autologous materials, and xenografts in bone regeneration, particularly in dental treatments such as alveolar ridge preservation, mandibular osteonecrosis, and peri-implantitis. Materials and Methods. A systematic literature search was conducted using databases like PubMed, Scopus, and Web of Science, with keywords such as “bone regeneration” and “CGF” from 2014 to 2024. Only English-language clinical studies involving human subjects were included. A total of 10 studies were selected for qualitative analysis. Data were processed through multiple stages, including title and abstract screening and full-text evaluation. Conclusion: The findings of the reviewed studies underscore the potential of the CGF in enhancing bone regeneration through stimulating cell proliferation, angiogenesis, and extracellular matrix mineralization. Autologous materials have also demonstrated promising results due to their biocompatibility and capacity for seamless integration with natural bone tissue. When combined with xenografts, these materials show synergistic effects in improving bone quantity and quality, which are crucial for dental implant success. Future research should focus on direct comparisons of different techniques, the optimization of protocols, and broader applications beyond dental medicine. The integration of CGFs and autologous materials into routine clinical practice represents a significant advancement in regenerative dental medicine, with the potential for improved patient outcomes and satisfaction.

## 1. Introduction

Bone regeneration plays a vital role in modern dental medicine, particularly in managing challenging conditions like alveolar ridge preservation, mandibular osteonecrosis, and peri-implantitis. Recent innovations, including concentrated growth factors (CGFs), autologous materials, and xenografts, have shown great promise in improving clinical outcomes [1,2,3,4,5]. These approaches not only address the inherent challenges of bone restoration but also offer exciting opportunities to enhance the quality and sustainability of dental treatments [6,7,8,9]. CGFs have gained significant attention for their biological versatility [10,11,12,13,14]. Enriched with key growth factors such as the platelet-derived growth factor (PDGF), the epidermal growth factor (EGF), and the vascular endothelial growth factor (VEGF), CGFs actively stimulate cellular proliferation, encourage the formation of new blood vessels, and promote the mineralization of the extracellular matrix [15,16,17,18,19]. These processes are essential for effective bone healing and regeneration [20,21,22,23]. Clinically, the CGF has proven highly effective in various scenarios, such as preserving alveolar ridges and managing the medication-related osteonecrosis of the jaw (MRONJ), establishing its value as a critical tool in regenerative dentistry [24,25,26]. Similarly, the use of autologous materials like dentin granules has shown remarkable potential due to their biocompatibility and ability to integrate seamlessly with natural bone [27,28,29,30,31]. These materials not only minimize risks associated with foreign grafts but also support robust bone formation by gradually releasing growth factors and activating key cellular pathways for osteogenesis [32,33,34,35,36]. When used in combination with xenografts, these materials provide a synergistic effect, further enhancing bone regeneration [37,38,39]. The application of these biologically active materials in clinical settings is supported by extensive research demonstrating their ability to improve both bone quality and volume—two essential factors for successful dental implant placement [40,41,42,43,44]. Maintaining the structural integrity of the alveolar ridge and minimizing complications after surgery are crucial for long-term outcomes [45,46,47]. As studies continue to uncover the underlying mechanisms driving these regenerative processes, the potential for broader applications in dental medicine and other areas of regenerative healthcare becomes increasingly apparent [48,49,50,51]. This article provides a thorough review of the clinical applications, biological mechanisms, and future possibilities for CGFs, autologous materials, and associated grafting techniques. In this review, we selected CGFs, autologous materials, and xenografts as the focus of analysis due to their distinct but complementary roles in bone regeneration. The CGF, derived from the patient’s own blood, offers a biologically active solution that enhances tissue repair and regeneration through their high concentration of growth factors. On the other hand, xenografts provide a structural scaffold that mimics the mineralized matrix of human bone, supporting osteoconduction and integration into the defect site. This study aims to highlight their individual contributions and potential synergistic effects, providing a comprehensive understanding of their clinical relevance and underlying biological mechanisms. By integrating findings from recent scientific studies, we aim to highlight the transformative potential of these innovations in advancing bone regeneration and improving outcomes for patients with complex dental conditions.

## 2. Materials and Methods

### 2.1. Processing Searches

We looked through three databases using the keywords “bone regeneration AND cgf” to locate studies that addressed this subject. Only English-language articles were considered, and the search was restricted to the previous ten years (2014–2024). Papers that met the following inclusion criteria were double-blindly selected by the reviewers: (1) publications that involve human subjects and (2) clinical research, case studies, or randomized controlled trials. Reviews and meta-analyses, research on animal models, and in vitro experiments fulfilled the exclusion criteria; English studies and papers lacking free full text were not included. The PROSPERO temporary registration code of this systematic review is ID 574282.

### 2.2. Data Processing

As part of the screening procedure, which comprised going over the article titles and abstracts chosen in the previous identification step, the full texts of the publications that had previously been included were read and any that did not suit the topics investigated were excluded. After the reviewers had discussed the selected papers, a third reviewer (FI) was consulted in cases of disagreement.

### 2.3. Quality Assessment

Using ROBINS, a method designed to evaluate the risk of bias in the findings of non-randomized studies that compare the health effects of two or more interventions, two reviewers, V.C. and C.C., evaluated the quality of the included publications. Each of the seven assessed points was given a bias level. If there was a dispute, a third reviewer, F.I., was consulted until a consensus was established.

## 3. Results

**Figure 1:**
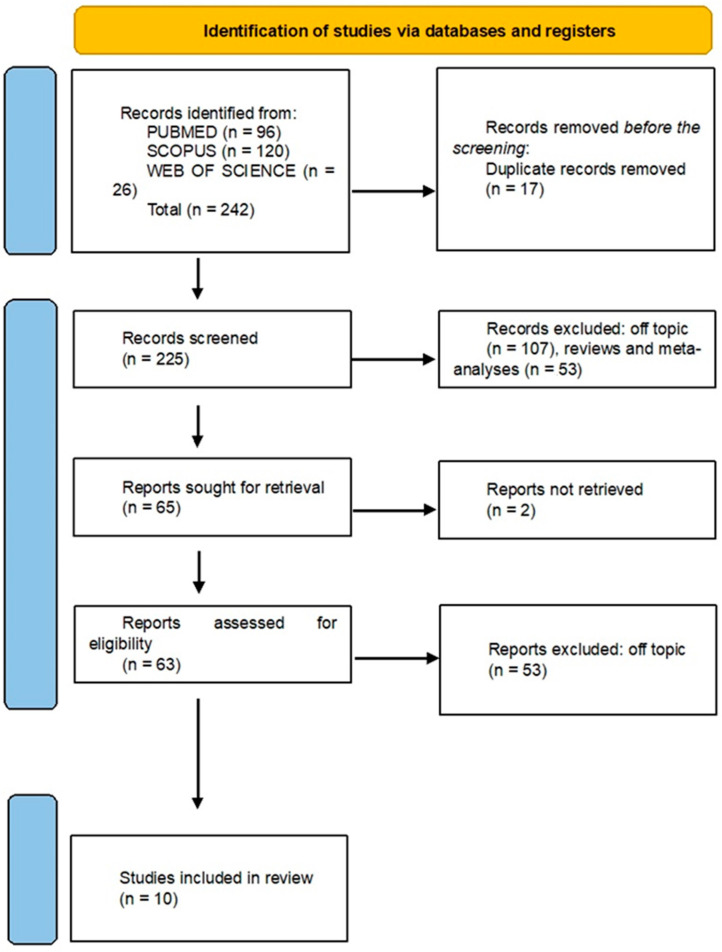
PRISMA flowchart.

Three databases were searched, yielding 242 publications: Pubmed (96), Web of Science (26), and Scopus (120). After 17 duplicate entries were removed, 225 records were screened for titles and abstracts, which resulted in the removal of 160 articles. Following a full-text review, 53 papers were excluded for failing to meet inclusion criteria, while 2 articles could not be located. Ten publications in all were ultimately determined to be suitable for qualitative analysis (Table 1). The selection process is summarized in Figure 1.

### 3.1. Quality Assessment and Risk of Bias of Included Articles

**Figure 2:**
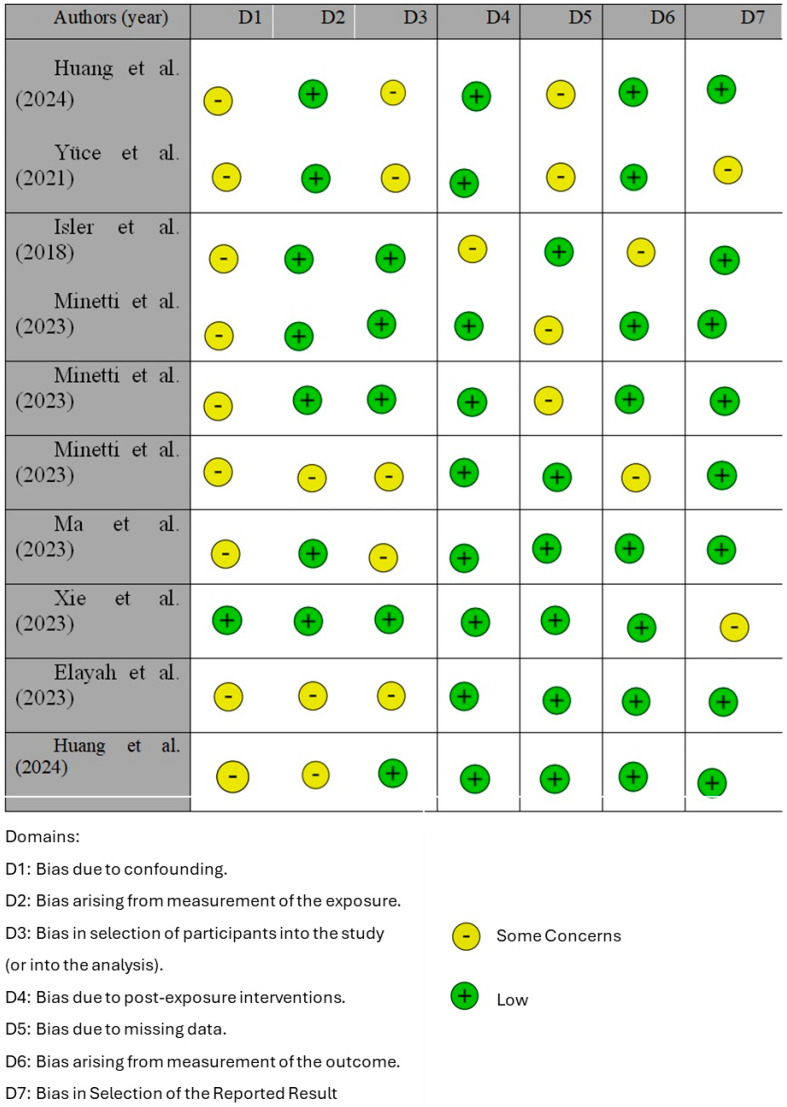
Risk of bias [8,12,17,21,27,37,52,53,54,55].

Figure 2 reports the risk of bias across the included studies, evaluated using the ROBINS tool. Overall, the studies exhibit a generally low risk of bias, though a few areas of concern were identified. Bias due to confounding (D1): While most studies effectively managed confounding factors (e.g., Xie et al., 2023, and Isler et al., 2018) [12,53], some, like Huang et al. (2024) and Ma et al. (2023) [27,55], showed concerns due to incomplete adjustments for potential confounders. For instance, baseline characteristics were not consistently reported, which could affect the comparability between study groups. Bias arising from the measurement of exposure (D2): This domain was well-handled by most studies. However, Huang et al. (2024) [27] raised moderate concerns due to ambiguities in the methods used to assess exposure, potentially impacting reproducibility. Bias in the selection of participants into the study (D3): Participant selection was robust in most studies, as seen in Elayah et al. (2023) [8]. However, smaller sample sizes in studies like Yüce et al. (2021) [52] introduced potential selection bias, reducing the generalizability of the findings. Bias due to post-exposure interventions (D4): Most studies exhibited low risks in this domain, as protocols were consistently followed. For example, Isler et al. (2018) [53] demonstrated clear and controlled intervention procedures, minimizing variability. Bias due to missing data (D5): While missing data were generally minimal, a few studies (e.g., Minetti et al., 2023) [21] faced challenges with incomplete follow-ups, which could have influenced the reported outcomes. Bias arising from the measurement of the outcome (D6): This was a source of concern in some studies, such as Ma et al. (2023) [55], where inconsistencies in the tools or timing of outcome assessments were noted. Such issues could reduce the reliability of the findings. Bias in the selection of the reported results (D7): Most studies demonstrated transparent reporting, with Huang et al. (2024) [27] providing a clear and detailed presentation of results. However, a few studies exhibited selective reporting, as noted in the variability of outcomes presented in Yüce et al. (2021) [52].

In conclusion, while the included studies are generally of high quality, areas requiring improvement include better control of confounding variables, more rigorous outcome measurement protocols, and enhanced strategies to minimize missing data. These findings, summarized in Figure 2, underscore the need for ongoing methodological refinement to further strengthen the evidence base.

### 3.2. Results and Comparative Analysis

The studies included in this review provide a diverse range of insights into the clinical applications of the CGF, emphasizing both its advantages and limitations in bone regeneration. Collectively, they suggest that the CGF holds promise as a versatile autologous material that can enhance healing, reduce postoperative complications, and improve bone formation. Clinical trials, such as those by Huang et al. (2018) and Ma et al. (2023) [37,55], demonstrate consistent benefits of the CGF in terms of reducing postoperative pain and promoting better bone preservation compared to controls. Similarly, studies like Elayah et al. (2023) [8] highlight the CGF’s ability to maintain alveolar ridge dimensions more effectively than conventional treatments. However, results regarding other parameters, such as swelling and bone density, are less consistent, suggesting variability in its effectiveness depending on the clinical context. When compared to other regenerative approaches, such as collagen membranes or mixed grafts, the CGF appears to offer comparable or superior outcomes in some parameters, such as bone augmentation and reduced postoperative discomfort (Xie et al., 2023) [12]. However, studies like Isler et al. (2018) [53] indicate that in certain applications, alternative materials may achieve better results, particularly in terms of clinical attachment levels and probing depth.

### 3.3. Clinical Significance and Limitations

The primary advantage of the CGF lies in its autologous nature, which eliminates immunogenicity and promotes natural healing processes. This aligns with the findings of multiple studies that report improved clinical and radiographic outcomes without significant adverse effects. Nonetheless, some studies, such as Yüce et al. (2021) [52], show limitations in statistical significance, which may stem from small sample sizes or variability in patient populations. A critical limitation observed across studies is the lack of long-term follow-up data to assess the durability of CGF-mediated regeneration. Additionally, the effectiveness of CGFs can vary based on the specific application, as highlighted by mixed results in studies examining its use in socket preservation and peri-implantitis treatment.

This analysis underscores the need for further high-quality, large-scale studies to fully establish the clinical potential and limitations of CGFs in bone regeneration. While current evidence supports its benefits in certain contexts, its comparative effectiveness and long-term outcomes remain areas for further investigation.

## 4. Discussion

The ten scientific articles analyzed provide an in-depth perspective on the use of CGFs, autologous materials, and xenografts in bone regeneration and the treatment of complex dental conditions. These studies address critical topics such as alveolar ridge preservation, mandibular osteonecrosis, and peri-implantitis, emphasizing the importance of innovative approaches in clinical practice.

### 4.1. Effectiveness of CGFs

CGFs have emerged as valuable tools for bone regeneration. Due to their high concentration of growth factors, CGFs play pivotal roles in various biological processes. For instance, they contain factors like the PDGF and the EGF, which are known to stimulate the proliferation of mesenchymal and osteogenic cells [56,57,58]. This is crucial for the creation of new bone tissue and the restoration of bone function. In their split-mouth randomized controlled trial, Elayah et al. demonstrated that CGFs significantly improved alveolar ridge preservation after dental extractions [50]. Similarly, Ma et al. conducted a prospective randomized controlled study showing the beneficial impact of CGFs on ridge preservation in posterior tooth extraction sites [55]. Furthermore, Yüce et al. explored the effectiveness of CGFs in the treatment of MRONJ in osteoporosis patients, showing a significant reduction in postoperative complications [52]. Additionally, CGFs promote angiogenesis—the formation of new blood vessels—which is essential for supplying nutrients and oxygen to regenerated bone tissues [5,59,60]. Factors like the VEGF present in CGFs are instrumental in this process [61,62,63,64,65]. Lastly, CGFs facilitate extracellular matrix mineralization, a necessary step for forming healthy, functional bones. These biomolecular mechanisms underscore the importance of CGFs in bone regeneration and tissue healing [66,67,68,69,70].

### 4.2. Bone Quality and Quantity

Numerous studies have demonstrated that the CGF significantly improves the quality and quantity of preserved bone [71,72,73,74]. CGF treatments have been shown to reduce bone loss in dental extraction sites, maintaining bone height and volume—critical factors for the success of dental implants [75,76,77,78]. A healthy alveolar ridge is essential for implant placement, and the results suggest that CGFs contribute to this goal [79,80,81,82]. For example, Yu Xie et al. conducted a randomized controlled clinical study that highlighted the effectiveness of sticky bone combined with CGFs in horizontal alveolar ridge augmentation for anterior teeth [12]. In another study, Huang et al. compared the osteogenic effects of the CGF with acellular dermal matrix, showing the superiority of the CGF in promoting new bone formation [37]. Additionally, Huang et al. conducted a split-mouth, randomized double-blind trial showing that the CGF significantly reduced postoperative complications in impacted third molar surgeries, emphasizing its role in clinical recovery [27].

### 4.3. Autologous Materials

Another important focus is the use of autologous materials, such as processed tooth granules. These materials offer several advantages, including optimal biocompatibility [83,84,85]. As autologous products, they minimize the risk of rejection or adverse reactions associated with foreign materials [86]. Additionally, tooth granules exhibit a favorable particle size distribution, depending on time and equipment, crucial for ensuring proper osseointegration and promoting bone growth [87,88,89,90,91]. E. Minetti et al. explored the dimensional characteristics of Tooth Transformer^®^ granules, emphasizing their potential as a regenerative material [21]. Furthermore, Elio Minetti et al. demonstrated in a pilot study that combining dentin granules with xenograft materials for socket preservation yielded promising results in clinical applications [54].

### 4.4. Biomolecular Mechanisms

The mechanisms by which CGFs and autologous materials exert their positive effects are multifaceted [92,93,94,95]. For example, CGFs release growth factors gradually, providing prolonged biological support that enhances regenerative responses at the bone site [96,97,98]. Moreover, these growth factors activate signaling pathways that promote stem cell differentiation into mature bone cells, essential for new bone formation [99,100,101]. Elio Minetti et al. further explored these mechanisms in their case series study of socket preservation using tooth grafts, providing insights into the biomolecular pathways involved [47]. Additionally, Yüce et al. showed that CGF-based treatments not only improved clinical outcomes but also reduced complications in challenging cases like MRONJ [52].

### 4.5. Clinical Implications

The findings of these studies have significant clinical implications. Adopting techniques that utilize CGFs and autologous materials can improve clinical outcomes in both the short and long term [102,103,104,105,106]. For instance, S. C. Isler et al. demonstrated in a 12-month randomized clinical trial that CGF-based techniques were highly effective in treating peri-implantitis compared to traditional collagen membranes [53].

The use of CGFs and autologous materials represents a promising approach to bone regeneration in dentistry [107,108,109,110,111]. These studies provide robust evidence supporting innovative techniques that can improve both the quality and quantity of preserved bone [112,113,114,115,116]. Continued research is essential to confirm these findings and explore additional clinical applications [117,118,119,120,121]. Despite the promising results, the studies presented have some limitations, including small sample sizes in pilot trials and the need for long-term follow-up to evaluate the sustainability of outcomes [122,123,124,125,126]. Larger, controlled studies are essential to validate these approaches and further explore the underlying biomolecular mechanisms [127,128,129,130,131]. Future research could focus on comparing different grafting techniques and materials to identify the optimal method for specific clinical scenarios, developing standardized protocols for preparing and applying CGFs and autologous materials and exploring the use of these techniques in other fields of regenerative medicine and various pathological conditions [132,133,134,135,136]. Integrating these innovative approaches into daily clinical practice could represent a significant advancement in dental medicine, leading to better outcomes and increased patient satisfaction [137,138,139,140].

While this review elucidates the biological mechanisms and clinical applications of concentrated growth factors (CGFs), a more comprehensive comparison of existing research findings offers valuable additional insights. For instance, Ma et al. demonstrated the efficacy of CGFs in ridge preservation, reporting significantly superior outcomes in bone volume retention when compared to the use of xenografts alone. In contrast, Elayah et al. observed no significant differences when the CGF was combined with autologous materials, suggesting that the choice of adjunctive material plays a crucial role in determining treatment outcomes.

Several factors may contribute to these observed discrepancies. Patient-specific variables, including age, systemic conditions (e.g., diabetes), and smoking habits, are well-documented determinants of bone regeneration and healing processes. Additionally, variations in CGF preparation protocols, such as differences in centrifugation speed and duration, can result in inconsistent concentrations of growth factors, thereby affecting clinical efficacy. Methodological inconsistencies, such as variability in follow-up durations and outcome assessment criteria, further complicate the ability to make direct comparisons between studies.

Finally, the current body of literature is constrained by small sample sizes and a lack of standardized reporting practices, which limit the robustness of conclusions. To overcome these challenges, standardized CGF preparation protocols and well-designed, large-scale randomized controlled trials are imperative to validate the clinical benefits and establish clearer evidence.

The continuous evolution of these technologies offers new opportunities to enhance care quality and treatment efficacy, contributing to safer and more effective clinical practice [141,142,143,144].

## 5. Conclusions

Bone regeneration represents a milestone in modern dental medicine, especially in addressing complex conditions such as alveolar process preservation, mandibular osteonecrosis, and peri-implantitis. Recent innovations, such as CGFs and autologous materials, offer extraordinary opportunities to improve clinical outcomes, ensuring more effective and sustainable treatments. The studies analyzed clearly demonstrate the potential of CGFs in stimulating the cell proliferation, angiogenesis, and mineralization of the extracellular matrix. These processes are crucial for successful bone regeneration. In parallel, autologous materials, such as dentin granules, emerge as biocompatible solutions that integrate seamlessly with natural bone tissue, reducing the risks associated with foreign materials. In particular, the combined efficacy of CGFs and xenografts has shown synergistic results, increasing the quantity and quality of regenerated bone tissue. This evidence is corroborated by significant improvements in the preservation of bone height and volume, which are crucial factors for the success of dental implants. However, limitations persist, including small samples and the need for longitudinal studies to confirm the long-term sustainability of these interventions. Future research should focus on direct comparisons between techniques, the optimization of material preparation protocols, and expanded applications to extra-dental pathologies. The integration of CGFs and autologous materials into daily clinical practice represents a significant advance in regenerative dental medicine. Such approaches not only improve the quality of care but also increase patient satisfaction, opening new perspectives for safe, effective, and durable treatment.

## Figures and Tables

**Table 1:** Featured research in the qualitative analysis and their characteristics.

Authors	Type of the Study	Aim of the Study	Materials and Methods	Results
Huang et al. (2018) [37]	Split-mouth randomized double-blind clinical trial	To evaluate the effectiveness of CGF in reducing postoperative complications after impacted third molar extraction.	A total of 25 patients with bilaterally impacted third molars. CGF was applied on one side, while the other side served as a control. Pain, swelling, and bone healing were assessed using CBCT.	Significant reduction in pain on the 3rd and 7th postoperative days in CGF sites compared to controls. No significant differences in swelling or bone healing between groups.
Yüce et al. (2021) [52]	Randomized Controlled Trial	To evaluate the effectiveness of concentrated growth factor (CGF) in the healing process of osteoporotic patients with MRONJ	A total of 28 elderly women with osteoporosis and MRONJ, divided into two groups: one treated with CGF and primary closure, the other with primary closure only. Postoperative analysis conducted over 6 months.	Complete healing in 19 out of 28 patients. The CGF group showed less bone exposure and infections, but results were not statistically significant.
Isler et al. (2018) [53]	A 12-month randomized clinical trial	To evaluate the clinical and radiographic outcomes of regenerative surgical treatment for peri-implantitis using CGF or collagen membranes.	A total of 52 patients with peri-implantitis were treated using bone substitutes combined with either collagen membranes or concentrated growth factors. Clinical and radiographic evaluations were conducted at baseline, 6, and 12 months.	Both treatment methods led to significant improvements in clinical and radiographic outcomes. At 12 months, collagen membranes showed better results in probing depth and clinical attachment level.
Minetti et al. (2023) [21]	Case Series Study	To assess the effectiveness of socket preservation using autologous tooth grafts.	A total of 20 socket preservation procedures with 18-month follow-up. Histological evaluation during implant placement.	Significant bone regeneration with uniform structure and no inflammation. Histomorphometric analysis shows promising results; further research needed for long-term outcomes.
Minetti et al. (2023) [54]	Pilot Study	To analyze mixed graft materials (50% dentin + 50% xenograft) for socket preservation.	Seven socket preservation surgeries with histological analysis at 4 and 8 months.	New bone formation at 29.03% (4 months) and 34.11% (8 months). Different absorption rates: dentin 71–90%; xenograft 6–26%. Dentin resorption increases new bone formation.
Minetti et al. (2023) [17]	Observational Study	To evaluate the granule size of bone graft materials from Tooth Transformer^®^ for osteogenesis.	Laser analysis of granules produced by Tooth Transformer^®^ device.	A total of 85% of granules were 100–1000 μm, aligning with literature recommendations for osteogenesis and bone regeneration.
Ma et al. (2023) [55]	Randomized Controlled Trial	To evaluate the impact of CGF on alveolar ridge preservation post-extraction.	A total of 50 patients randomized to CGF or control groups; healing scores, CBCT, and computerized microtomography analyses were performed.	CGF improves healing scores, reduces vertical and horizontal bone resorption, and enhances new bone formation compared to controls.
Xie et al. (2023) [12]	Randomized Controlled Trial	To evaluate sticky bone combined with CGF for anterior alveolar ridge augmentation.	A total of 28 patients randomized to sticky bone with CGF or saline-mixed bone powders; CBCT analysis and VAS scores.	Sticky bone with CGF improves bone augmentation (72% vs. 57% volume conversion) and reduces pain (lower VAS scores).
Elayah et al. (2023) [8]	Randomized Controlled Trial	To assess the efficacy of CGF in ridge preservation following lower third molar extraction.	A total of 60 sites in 30 patients compared CGF-treated sockets to controls; CBCT and histological analysis.	CGF-treated sockets show greater bone height, width, and density. Improved periodontal pocket reduction and bone preservation.
Huang et al. (2024) [27]	Randomized, Double-Blind, Split-Mouth Trial	To evaluate the effect of concentrated growth factor (CGF) in reducing postoperative complications after mandibular third molar extractions.	A total of 25 patients with bilaterally impacted third molars (50 extraction sites) were included. Each patient acted as their own control. CGF was placed in one extraction socket, while the other was sutured without CGF. Pain, swelling, and bone healing were assessed postoperatively.	Significant pain reduction was observed on the 3rd and 7th postoperative days in the CGF group. No significant differences were found in facial swelling or bone healing between the CGF and control groups. No adverse effects were reported.

## Data Availability

No new data were created or analyzed in this study. Data sharing is not applicable to this article.

## Abbreviations

CGF	concentrated growth factor
EGF	epidermal growth factor
MRONJ	medication-related osteonecrosis of the jaw
PDGF	platelet-derived growth factor
VEGF	vascular endothelial growth factor
